# APPLAUSE: Automatic Prediction of PLAcental health via U-net Segmentation and statistical Evaluation

**DOI:** 10.1016/j.media.2021.102145

**Published:** 2021-08

**Authors:** Maximilian Pietsch, Alison Ho, Alessia Bardanzellu, Aya Mutaz Ahmad Zeidan, Lucy C. Chappell, Joseph V. Hajnal, Mary Rutherford, Jana Hutter

**Affiliations:** a Centre for Medical Engineering, King’s College London, London, UK; b Centre for the Developing Brain, King’s College London, London, UK; c Department of Women and Children’s Health, King’s College London, London, UK

**Keywords:** Placental MRI, Relaxometry, Acquisition, Pre-eclampsia, U-Net, Segmentation, Regression, Gaussian process

## Abstract

*Purpose:*Artificial-intelligence population-based automated quantification of placental maturation and health from a rapid functional Magnetic Resonance scan. The placenta plays a crucial role for any successful human pregnancy. Deviations from the normal dynamic maturation throughout gestation are closely linked to major pregnancy complications. Antenatal assessment in-vivo using T2* relaxometry has shown great promise to inform management and possible interventions but clinical translation is hampered by time consuming manual segmentation and analysis techniques based on comparison against normative curves over gestation.

*Methods:*This study proposes a fully automatic pipeline to predict the biological age and health of the placenta based on a free-breathing rapid (sub-30 second) T2* scan in two steps: Automatic segmentation using a U-Net and a Gaussian process regression model to characterize placental maturation and health. These are trained and evaluated on 108 3T MRI placental data sets, the evaluation included 20 high-risk pregnancies diagnosed with pre-eclampsia and/or fetal growth restriction. An independent cohort imaged at 1.5 T is used to assess the generalization of the training and evaluation pipeline.

*Results:* Across low- and high-risk groups, automatic segmentation performs worse than inter-rater performance (mean Dice coefficients of 0.58 and 0.68, respectively) but is sufficient for estimating placental mean T2* (0.986 Pearson Correlation Coefficient). The placental health prediction achieves an excellent ability to differentiate cases of placental insufficiency between 27 and 33 weeks. High abnormality scores correlate with low birth weight, premature birth and histopathological findings. Retrospective application on a different cohort imaged at 1.5 T illustrates the ability for direct clinical translation.

*Conclusion:*The presented automatic pipeline facilitates a fast, robust and reliable prediction of placental maturation. It yields human-interpretable and verifiable intermediate results and quantifies uncertainties on the cohort-level and for individual predictions. The proposed machine-learning pipeline runs in close to real-time and, deployed in clinical settings, has the potential to become a cornerstone of diagnosis and intervention of placental insufficiency. APPLAUSE generalizes to an independent cohort imaged at 1.5 T, demonstrating robustness to different operational and clinical environments.

## Highlights

• Automatic placental health prediction based on a 30 s MRI scan can identify early placental insufficiency (AUC = 0.95).
• An abnormal automatic maturation marker is associated with low birth weight centile and premature birth.
• The automatic maturation marker correlates with presence of maternal vascular malperfusion in the abnormal cohort.
• Explicit uncertainty modeling and separation into human-interpretable steps facilitates clinical use and future extensions.

## Graphical abstract

**Figure:**
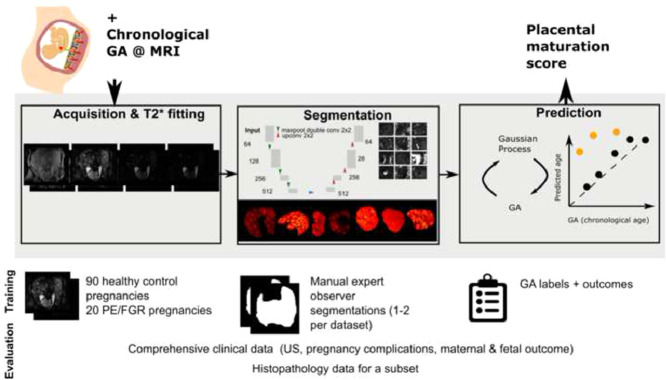

## 1 Introduction

### 1.1 Placental maturation

The human placenta is key for any successful human pregnancy. It grows and changes across gestation to adapt to the increasing demands of the fetus. To this end the increased transfer of nutrients and oxygen from the maternal circulation to the fetal circulation is of key importance. This exchange process, occurring in 20–40 functional units or *lobules* across the placenta, relies on sufficient inflow from the maternal spiral arteries, through the fetal vasculature in the villous trees, into the umbilical vein, to match fetal demand (Burton, Woods, Jauniaux, Kingdom, 2009, Acharya, Sonesson, Flo, Räsänen, Odibo, 2016). This low resistance system relies on ongoing combined angiogenesis and villous transformation with initial sprouting and branching followed by stromal reduction to increase the vasculo-syncitial membrane area to maximise oxygen and nutrient transfer (Demir et al., 2007). As the placenta ages, there is increased deposition of calcium within the lobules as well as deposition of fibrin largely within the septa between the lobules. The fibrin deposition contributes to the lobulated appearance on imaging, seen as low signal intensity on T2-weighted images, and the calcium deposition to an increase in granularity within the placenta as a whole (Andescavage et al., 2015). This process of placental aging occurs normally in accord with gestational age (GA) and the associated changes are not pathological. However, accelerated aging of the placenta, as documented on histopathology, has been associated with increased risk of placental failure, fetal growth restriction, pre-eclampsia (PE) and unexplained late stillbirth (Stallmach, Hebisch, Meier, Dudenhausen, Vogel, 2001, Benton, Leavey, Grynspan, Cox, Bainbridge, 2018). In contrast, delayed maturation of the placenta is associated with gestational diabetes and chromosomal abnormalities (Benton, Leavey, Grynspan, Cox, Bainbridge, 2018, Turowski, Vogel, 2018, Stallmach, Hebisch, Meier, Dudenhausen, Vogel, 2001). Thus, advancing our understanding of placental maturation and identifying tools to describe and quantify this process are vital to help improve early detection of placental failure. Functional MR imaging could provide a quantifiable marker of placental aging and could facilitate identification of accelerated aging or of a delayed trajectory in individual placentas. Clinically, the ability to identify cases of placental insufficiency antenatally, prior to post-delivery histopathological assessment, is key to enable close monitoring and potential interventions. Recent research endeavours such as the Human Placenta Project therefore focused on imaging techniques to provide such an early assessment. Current clinical screening and monitoring for placental insufficiency includes ultrasound and especially Doppler ultrasound assessment of the blood flow in the uterine and umbilical arteries. These do however not provide direct insights into the placental tissue and function and, as a consequence, alteration in these values often indicates a later stage of placental insufficiency. MRI techniques in contrast allow using bespoke contrasts to capture placental tissue properties.

### 1.2 State of the art

Placental development over gestation has been recently studied with T2* relaxometry (Sørensen, Peters, Fründ, Lingman, Christiansen, Uldbjerg, 2013, Ingram, Morris, Naish, Myers, Johnstone, 2017, Sinding, Peters, Frøkjaer, Christiansen, Petersen, Uldbjerg, Sørensen, 2016, Slator, Hutter, Palombo, Jackson, Ho, Panagiotaki, Chappell, Rutherford, Hajnal, Alexander, 2019, Hutter, Slator, Jackson, Gomes, Ho, Story, O’Muircheartaigh, Teixeira, Chappell, Alexander, Rutherford, Hajnal, 2018, Sørensen, Hutter, Seed, Grant, Gowland, 2020, Turk, Luo, Copeland, Restrepo, Borjan Gagoski, Wald, Adalsteinsson, Roberts, Golland, Grant, Barth Jr, 2018, Dellschaft, Hutchinson, Shah, Jones, Bradley, Leach, Platt, Bowtell, Gowland, 2020). The T2* values can be linked to the concentration of deoxygenated hemoglobin via the BOLD effect and thus provide both an ability to visually inspect and quantify function in spatial maps cross-sectionally and over gestation. Many recent studies have employed these techniques to study the placenta in high-risk pregnancies compared to control low-risk pregnancies with normal outcomes. The majority of studies compare placental mean T2* values between controls and high-risk cohorts.

*Segmentation* A crucial step for any quantitative, or indeed, qualitative assessment is the detection and delineation of the placenta. The heterogeneous placental shape and variation of its location on images that typically cover the entire uterus, including maternal, fetal and placental tissue as well as amniotic fluid, hampers this step. For current published studies, the obtained T2* maps are manually segmented, either on selected slices (Sinding, Peters, Frøkjaer, Christiansen, Petersen, Uldbjerg, Sørensen, 2016, Sørensen, Peters, Fründ, Lingman, Christiansen, Uldbjerg, 2013) or across the entire placental volume (Abaci Turk, Abulnaga, Luo, Stout, Feldman, Turk, Gagoski, Wald, Adalsteinsson, Roberts, Bibbo, Robinson, Golland, Grant, Barth, 2020, Hutter, Slator, Jackson, Gomes, Ho, Story, O’Muircheartaigh, Teixeira, Chappell, Alexander, Rutherford, Hajnal, 2018, Slator, Hutter, Palombo, Jackson, Ho, Panagiotaki, Chappell, Rutherford, Hajnal, Alexander, 2019). First attempts to automate placental segmentation have already been undertaken for anatomical placental scans. The interactive Slic-Seg approach was proposed using random forests within slices (Wang et al., 2015) and subsequently improved using probability-based 4D Graph Cuts and deep learning. The latter utilizes user interactions after an initial convolutional neural network to refine the segmentation with a second convolutional neural network. Fully automatic frameworks were proposed using 3D multi-scale convolution neural networks to identify the area of interest, followed by 3D dense conditional random fields (Alansary et al., 2016) and U-net based segmentation (Han et al., 2019). Finally, a technique combining motion correction, segmentation and shape extraction has been proposed (Miao et al., 2017). Similarly, in 3D placental US imaging, seed-based random walker techniques (Stevenson et al., 2015) and U-nets have been successfully employed for volumetric segmentations. However, for functional imaging data, to date only manual placental segmentations have been used (Abaci Turk, Abulnaga, Luo, Stout, Feldman, Turk, Gagoski, Wald, Adalsteinsson, Roberts, Bibbo, Robinson, Golland, Grant, Barth, 2020, Sinding, Peters, Frøkjaer, Christiansen, Petersen, Uldbjerg, Sørensen, 2016, Sinding, Peters, Frøkjær, Christiansen, Petersen, Uldbjerg, Sørensen, 2017, Sørensen, Peters, Fründ, Lingman, Christiansen, Uldbjerg, 2013, Hutter, Slator, Jackson, Gomes, Ho, Story, O’Muircheartaigh, Teixeira, Chappell, Alexander, Rutherford, Hajnal, 2018, Slator, Hutter, Palombo, Jackson, Ho, Panagiotaki, Chappell, Rutherford, Hajnal, Alexander, 2019). Manual segmentations in functional placental data such as T2* maps remain a time consuming task with substantial disagreement even among expert annotators. This is due to the low Signal-to-Noise-Ratio, the complex and widely varying tissue characteristics, the often unclear boundary between uterine wall and placental tissue and the presence of maternal vessels and fetal surface vasculature.

*Disease progression using Gaussian processes* Conventionally, following segmentation, quantitative measures are obtained, most often the mean T2*, averaged across the entire placenta, which are assessed against “normative” curves over gestation obtained from longitudinal or cross-sectional studies (Sørensen, Peters, Fründ, Lingman, Christiansen, Uldbjerg, 2013, Sinding, Peters, Frøkjaer, Christiansen, Petersen, Uldbjerg, Sørensen, 2016, Sinding, Peters, Poulsen, Frøkjær, Christiansen, Petersen, Uldbjerg, Sørensen, 2018, Hutter, Slator, Jackson, Gomes, Ho, Story, O’Muircheartaigh, Teixeira, Chappell, Alexander, Rutherford, Hajnal, 2018, Armstrong, Liu, Martin, Masamed, Janzen, Wong, Chanlaw, Devaskar, Sung, Wu, 2018). Using a group of placentas of normal appearance, a normative curve and credibility intervals can be derived directly from the data using a Bayesian regression algorithm. Assuming abnormal placental development manifests as a mean T2* signal deviation, this can be used to estimate an abnormality score, taking the uncertainty of the model fit into account. We use Gaussian process regression (Rasmussen, 2004), a non-parametric algorithm that assigns a probability to each possible function describing the training data, and allows the calculation of normative curves (the mean of its posterior distribution) as well as credibility regions around these. This technique has been successfully used previously, for instance, in the setting of neuroimaging (Cole, Franke, Cole, Ritchie, Bastin, Valdés Hernández, Muñoz Maniega, Royle, Corley, Pattie, Harris, Zhang, Wray, Redmond, Marioni, Starr, Cox, Wardlaw, Sharp, Deary, 2018, Steffener, Habeck, O’Shea, Razlighi, Bherer, Stern, 2016). A key difference to these studies is however the inter-subject variability of placental shape, size and location which hinders the construction of atlases or standard planes. Hence, we use the scalar measures GA and mean T2* inside the automatically segmented placenta to estimate normal and abnormal maturation.

A placental age prediction model could be used to identify abnormal data via comparison of “predicted biological placental age” and chronological age. Similarly, we could predict mean T2* for a given GA and determine the difference between measured and expected values. We use an approach based on error-in-variables modelling, that takes uncertainties associated with both quantities into account to minimise prediction bias.

### 1.3 Contributions

This work seeks to establish a fully automatic pipeline using mean T2* measurements as a biomarker of placental maturation and health. The focus is on mean T2* as a tissue property to characterize normal maturation and abnormal tissue changes in-vivo, facilitating early assessment, monitoring and intervention planning for underlying clinical conditions. A data set of well characterized uncomplicated pregnancies as well as a range of complicated pregnancies allows us to study placental maturation in depth.

We aim to do this in three different ways:

1. Demonstrate the automated pipeline consisting of a sub-30 second MRI scan, an automated U-net based segmentation, and subsequent placental health estimation.
2. Demonstrate that the studied techniques can accurately capture the credibility interval of low-risk data which can be used to build a normative model of placental maturation in a cohort of well characterized uncomplicated pregnancies and to assess a cohort of pregnancies affected by pregnancy complications.
3. Demonstrate that the proposed concepts can be translated to MRI data with substantially different properties such as a reduced field strength (1.5 T instead of 3 T). For this, the overall pipeline developed for the training cohort was applied to the data acquired at 1.5 T with fixed hyperparameters but re-estimated U-net and Gaussian process regression model parameters.

## 2 Materials and methods

**Fig. 1:**
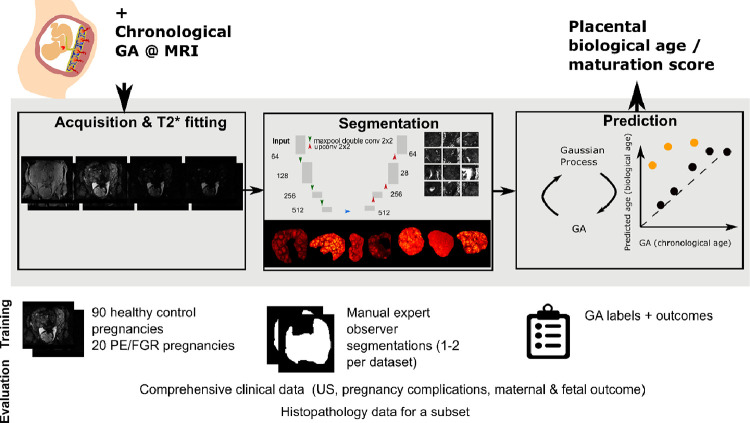
The proposed algorithm to obtain the biological age of the placenta to assess maturation is presented in the grey box together with input and output above, training data and evaluation data below.

The proposed pipeline is depicted in Fig. 1 consisting of the data acquisition, automatic segmentation used to calculate the placental mean T2* and the Gaussian process regression fit or prediction, which is used to characterize placental health. All parts will be discussed in detail in the following.

### 2.1 Data acquisition and preparation

MR imaging was performed on 110 women with singleton pregnancies between 18 and 40 weeks GA without contraindications to MRI as part of the Placental Imaging Project (https://placentaimagingproject.org/, REC 14/LO/1169) after informed consent was obtained. The scans were performed on a clinical 3T Philips Achieva (Best, The Netherlands) scanner using the 32-channel cardiac receiver coil. All women were scanned in the supine position with frequent monitoring of their heart rate, saturation and blood pressure throughout the scan. Dedicated padding was provided to increase maternal comfort and verbal communication was maintained. Clinical information and pregnancy outcome were obtained. For the fetus, this included gestation at birth, birth weight and birth weight centile calculated using the INTERGROWTH algorithm (Papageorghiou et al., 2014), Apgar score and admission to neonatal unit. Maternal age, Body Mass Index (BMI), clinical history, ethnicity and any diagnosis of PE, fetal growth restriction, hypertension, gestational diabetes mellitus and any other complications were recorded. Analysis of subsequent clinical outcome data allowed us to define a low-risk cohort and a high-risk cohort diagnosed with PE or growth restriction using the criteria outlined previously (Ho, Hutter, Jackson, Seed, McCabe, Al-Adnani, Marnerides, George, Story, Hajnal, Rutherford, Chappell, 2020, Figueras, Gratacós, 2014). As we require the low-risk cohort to yield normative values for the biological age prediction, we applied a fixed and conservative set of exclusion criteria. These included birth at GA between 37 and 41 weeks, birth weight centile between the 3rd and 97th centile, confirmation, that no diagnosis of PE, FGR, gestational hypertension or any other significant maternal complication was diagnosed, APGAR score at 10 minutes of at least 9 and no need for ventilation or intensive neonatal care.

The GA at scan was obtained based on the agreed expected date of delivery from ultrasound exams between 11 and 14 weeks of gestation. In addition, for 39 participants of the healthy and 17 participants from the high-risk cohort histopathological data were available. Macroscopic and microscopic evaluation of the placenta was performed and the presence of maternal vascular malperfusion and signs of chorioamnionitis were recorded. Details of these cohorts are given in Table 2 and graphically illustrated in Supplementary Figure 8.

**Table 1:** MRI parameters.

FOV [mm × mm]	340 × 400
**Resolution** [mm${}^{3}$]	3 × 3 × 3
**Slices**	24–86
**Orientation**	coronal
**TEs** [ms]	13.8, 70.4, 127, 183.6
**TR** [s]	12
**SENSE**	3
**Halfscan**	0.6
**TA** [s]	26

**Table 2:** Characteristics of the study participants. The cohort included low-risk pregnancies without evidence of PE, fetal growth restriction, Gestational Diabetes Mellitus or hypertension resulting in a live birth at 36 weeks or above with a birth weight between the 2nd and 98th centile and a high-risk cohort of pregnancies diagnosed with PE and or fetal growth restriction. Δ GA corresponds to the time between scan and delivery in weeks. For scatter plots see Figure 8.

	Low-risk	Placental insufficiency
**n**	90	20
**GA at MRI**	29.93±4.26	30.83±3.52
weeks		
**Maternal age**	33.9±3.54	32.52±6.69
years		
**BMI**	22.48±2.66	24.65±2.99
kg/m${}^{2}$		
**GA delivery**	40.09±1.23	34.07±3.27
weeks		
**Fetal sex**	40.09	34.07
% female		
**Birth weight**	3.41±0.43	1.77±0.7
kg		
**Birth weight**	55.72±27.28	13.45±18.39
centile		
Δ**GA**	10.06±4.46	3.23±3.27
weeks		

After initial fetal brain and whole uterus anatomical imaging, a map of the B0 field was acquired and image-based shimming was conducted. Then, a free-breathing multi-echo Gradient Echo technique with an Echo Planar Imaging read-out was performed. The parameters for this sequence are specified in Table 1. The local requirement is to keep the acoustic output of all sequences used for routine fetal imaging below 98 dB(A) which drives the choice of TEs and resolution. The total acquisition time was 26 seconds. All acquisitions were performed free-breathing to maximize maternal comfort and clinical applicability. Six data sets with either signs of sub-clinical contractions, seen as transient areas of low signal distorting the uterine wall, or containing unresolved fat artefacts were discarded. For four of these cases, a second, contraction-free scan was available in the same session and was used instead, resulting in a total final number of 108 scans considered. This data pre-selection step to identify placental contractions can be performed in less than one minute by trained placental analysts. Work on automatic identification based on 3D reconstruction is ongoing (Uus et al., 2020).

A mono-exponential decay model was fitted on a voxel-level using a Levenberg–Marquart algorithm with 50 iterations, initialised using the first echo time. Slices were fitted independently, and as all echos for one slice were acquired within 200ms, no motion was observed *within slices* obviating the need for motion correction. T2* values over 300 ms were clipped to limit partial volume effects from amniotic fluid close to the placental boundaries.

The placenta was manually outlined on all slices by one or two experienced placental analysts [AH and JH, clinician and imaging scientist with 4 years of experience] using the fitted T2* map and/or the image data corresponding to individual echo times. If two segmentations were available, one was randomly chosen and used for training and evaluation of the U-net.

### 2.2 Placental segmentation

The network architecture is based on the 2D U-net implementation *nnU-Net3* (Isensee et al., 2019). Training and inference were performed on 2D patches of 64x64 voxels. The data pre-processing included normalizing of the T2* maps by demeaning with the mean of the training dataset. For training, data augmentation in the form of spatial cropping, mirroring and rotations was applied using the *batchgenerator* framework^1^ (Isensee et al., 2020). The network is trained via stochastic gradient descent using the Adam optimizer (Kingma and Ba, 2015) with a learning rate of 0.0001 and batch size of five for 50,000 epochs using a binary cross entropy loss function. The training of the network utilizes 12 GB of VRAM and takes approximately 2 days on a Tesla P100 GPU on Google collaboratory.

*Training and test data for the segmentation* To test out-of-sample performance, five-fold stratified cross-validation was performed. All reported segmentation and hereof based T2* measurements were generated using the network for which these data were part of the held-out data. To avoid leakage between training and test data, participants were assigned exclusively to one of the partitions. Each training round was performed on 72 healthy low-risk cases and 16 high-risk abnormal cases, the test data consisting of 18 control and 4 high-risk cases. If two segmentations were available, one was chosen randomly to perform the training as specified above.

*Evaluation* Each network was evaluated on its corresponding test data. To match the training process, inference was performed on 64x64 patches of the validation set with patches chosen to overlap by 30%. The predicted probability was averaged across overlapping predictions and binarized to obtain the full 3D mask. The performance was evaluated with two metrics, first via direct evaluation of the segmentation using the Dice coefficient (Dice, 1945). Second, agreement between the mean T2* value between the manual and automatic segmented placental mean T2* results was evaluated on the test data using the Pearson Correlation Coefficient and Root Mean Square Error. Both metrics, mean T2* and the Dice coefficient were also evaluated to compare the two manual segmentations where both were available.

### 2.3 Unbiased placental health prediction

The final part of the pipeline consisted of a Gaussian process regression model that uses the mean T2* data generated via automated segmentations to capture normal maturation. Strict selection of normal, uncomplicated pregnancies based both on the information available at the time of the MRI as well as comprehensive clinical information from the remainder of the pregnancy and both neonatal and maternal outcome allowed us to us the chronological GA as a substitute for biological placental age.

*Biological age prediction* To predict biological placental age $t_{b}$ from the mean T2* value inside the automatically segmented placental mask ($\bar{T2^{*}}$), we assumed a probabilistic function $t_{b}=f\left(\bar{T2^{*}}\right)+\epsilon$ with independent and identically normally-distributed noise $\epsilon ∼N(0,\sigma_{\epsilon }^{2}1)$. We trained a Gaussian process regression model to approximate $f\left(x\right)∼\mathrm{GP}(\mu \left(x\right),k_{\theta }\left(x,x^{\prime }\right)),$ where μ(x) is the mean function and $k_{\Theta }$ the covariance function with hyperparameters θ. Given the relatively smooth and continuous relation of the data in a GA-vs-T2* plot, we chose μ to be constant and k to model a linear trend with local nonlinear deviation using a sum of dot product kernel and squared-exponential function kernel (length-scale $\leq 1\sigma (T2^{*})$) with an additive white noise kernel. Model hyperparameters were estimated by maximizing the log marginal likelihood, maximizing the probability of the training data conditioned on kernel hyperparameters. All fits were performed using *SciKitLearn* (Pedregosa et al., 2011). The performance was evaluated by calculating the mean squared error and Pearson correlation coefficient between biological GA ($t_{b}$) and predicted GA.

*Total least squares Gaussian process regression* In the ordinary least squares (OLS) regression model $t_{b}=f\left(\bar{T2^{*}}\right)+\epsilon ,$ it is assumed that the independent variable ($\bar{T2^{*}}$) is measured without error; all error is attributed to $t_{b}$. However, $\bar{T2^{*}}$ values are derived from noisy MRI measurements using imperfect segmentations and $\bar{T2^{*}}$ might be influenced by factors not related to age. Since biological age is known relatively precisely, the majority of the errors in the least squares fit could be associated with the independent variable. To obtain an unbiased estimate of placental health, an errors-in-variables model (Cheng, Van Ness, 1999, Chesher, 1991, Van Huffel, 2004, Schennach, 2016) that takes both sources of error into account is required. A commonly used errors-in-variables technique is Total Least Squares (TLS), which minimises the squared distance orthogonal to the fitted line or to the fitted (hyper-)plane in higher dimensions instead of along the direction of a single dependent variable. Different contributions to the expected residuals can be accounted for by scaling the data prior to fitting so that their expected errors are equal (Golub, Van Loan, 1980, Van Huffel, 2004).

To be able to use a Gaussian process regression fit, we transformed each of the $\left(t_{b},\bar{T2^{*}}\right)$ data points to a form that allowed fitting an approximately unbiased estimator using a least squares fit. The algorithm for projecting the data to learning an unbiased Gaussian process regression model is: First the age and mean T2* of the n measurements are stored in the columns of the 2×n data matrix X, which is demeaned and, as in the TLS approach, scaled according to the expected errors in the training set of both quantities $X_{s}=SX,$ with $S=\left[\begin{array}{cc} \left(1-E\left(t_{b}\right)\right)/\sigma_{\epsilon } & 0\\ 0 & 1-E\left(\bar{T2^{*}}\right)/\sigma_{\delta } \end{array}\right]$. Second, $X_{s}$ is rotated so that its rows represent the scaled data transformed onto the first and second principal axes of $X_{s}$: $X^{\prime }=U^{T}SX,$ where the columns of U hold the left singular vectors of $X_{s}$ computed via singular value decomposition $X_{s}=U\Sigma V^{T}$. Finally, the Gaussian process $f_{\Theta }$ is fitted to predict the second row of the projected data $X_{2,:}^{\prime }$ given the first row $X_{1,:}^{\prime }$.

The algorithm for calculating the mean of the predictive distribution of unseen data $\left(t_{b,u},\bar{T2_{u}^{*}}\right)$ consists of three steps. First, equivalently to $X^{\prime },$ the data matrix $Y=\left[\begin{array}{c} t_{b,u}\\ \bar{T2_{u}^{*}} \end{array}\right]$ is constructed, scaled and rotated $Y^{\prime }=U^{T}SY$. Second, the predictive distribution of the Gaussian process is queried at locations $Y_{1,:}^{\prime }$. Finally, the matrix $\hat{Y^{\prime }}=\left[\begin{array}{c} Y_{1,:}^{\prime }\\ f_{\Theta }\left(Y_{1,:}^{\prime }\right) \end{array}\right]$ that contains the query points and Gaussian process predictions in its rows is projected back to the space of X via the inverse projection $\hat{Y}=S^{-1}U\hat{Y^{\prime }}$.

*Training and test data for the prediction* We aim to evaluate the performance of the proposed pipeline on as much data as possible. Since the Gaussian process regression model relies on the automatic segmentation, we used the segmentations generated on the cross-validation test-set and the respective U-net models to predict placental masks. This allowed calculating unbiased mean T2* values for all images.

For training and evaluation of the Gaussian process based health prediction, data from the low-risk control cohort were split into a training and test set in the ratio 7:3. Only low-risk cases were chosen for training as only these allow the Gaussian process to accurately model normal maturation over gestation. The model was tested on the hold-out low-risk test data and the data from the high-risk cohort.

*Evaluation* A Gaussian process regression fit was performed to predict the first row of $X^{\prime },$ given the second. This allows capture of any potentially non-linear relations not accounted for by the first principal direction and to determine Z-scores and confidence regions using the Gaussian process posterior. Z-scores for unseen data can be estimated using the transformation matrices and Gaussian process posterior estimated from the training data. For visualisation, Gaussian process predictions can be transformed back and shown in the original space of X using the inverse projection $S^{-1}U$.

*Probability for accelerated aging* The posterior predictive distribution of a Gaussian process regression model can be used to describe the predictive distribution which allows estimating credibility intervals of the data. This allows the calculation of Z-scores for new observations. In our total least squares Gaussian process regression framework, Z-scores are calculated in the projected space ($Y^{\prime }$) and credibility intervals are transformed back to the measurement space via the inverse projection. The posterior distribution is conditional on the training data and on the hyperparameters but it assumes noiseless input data for inference. Given knowledge of the uncertainty associated with the input data, we can integrate over this input data distribution to estimate the true predictive distribution for noisy data. For new observations, we assume a multivariate normal noise model with covariance matrix $\text{diag}(\sigma_{\epsilon }^{2},\sigma_{\delta }^{2})$ and estimate the corresponding predictive distribution via Markov Chain Monte-Carlo sampling (10,000 samples). Conditional on the hyperparameters, training data, and input noise estimates, we can then calculate the expected probability that a placenta exhibits abnormally accelerated aging (Z<−3) from a single mean T2* measurement. To obtain a measure of the diagnostic ability to classify high-risk from low-risk placentas via the proposed method, we calculate the area under the curve (AUC) of the receiver operator characteristic curve.

*Data uncertainty estimate* For the total least squares fit, we estimate the errors associated with the two variables of interest. We model biological placental age using GA, which is not necessarily an accurate measure of development, as normal full-term gestation ranges from 37 to 42 weeks. Therefore, assuming normally-distributed errors, we expect the standard deviation of the biological age of the placenta $\sigma_{\epsilon }$ to be (42−37)/(2*1.96)≈1.3 weeks. We assume that the true not observable value of $\bar{T2^{*}}$ is corrupted by additive errors $\delta ∼N(0,\sigma_{\delta }^{2}1)$. With fixed $\sigma_{\epsilon },$ we estimate the 95% CI for $\bar{T2^{*}}$ from a point estimate in 1σ age proximity of the highest data density as 35ms, yielding $\sigma_{\delta }=8.7\text{ms},$ which is slightly larger than 7.4ms, the root mean squared error of mean T2* between the manual and automatic segmentations reported in Section 3.1.

### 2.4 Generalization and 1.5 T data

Summarizing, once trained, the complete algorithm for unseen data consists of the acquisition and mono-exponential fitting of multi-echo gradient echo data followed by segmentation via the trained U-net and finally estimation of the Z-score or accelerated aging probability using the Gaussian process regression model. The translation to a new scanner and MRI protocol requires retraining or fine-tuning the segmentation U-net and re-estimation of the Gaussian process parameters. Hence, translation requires a cohort of low-risk scans spanning the age-range of interest as well as manual segmentations for low-risk and, depending on image characteristics, high-risk groups.

To investigate the generalization of the method to other clinical cohorts and scanner environments, data were acquired on a clinical 1.5 T Philips Ingenia scanner in cohorts of women with low-risk and high-risk pregnancies. These cohorts were in general characterized by a higher BMI due to the larger bore size of the employed 1.5 T scanner. We note that increased maternal weight is associated with decreased image quality and increased maternal health risks including PE (Hennig et al., 2019). The high-risk 1.5 T cohort was uniquely composed of PE cases. A total of 42 data sets were acquired, including 36 control and 6 PE cases. The protocol was adapted as far as required for the different scanner conditions. Specifically, the inter-echo spacing was changed to adapt to the acoustic output of the 1.5 T scanner. The spatial resolution was slightly increased from 3 mm to 2.5 mm to comply with other ongoing projects. These data were processed and analysed using the pipeline developed for the 3 T data as described in the method section. The 1.5 T data consist of fewer measurements, hindering robust uncertainty estimation. Therefore, we pooled all low-risk data (training and test) to define four age bins containing at least four measurements within $\pm 1\sigma_{\epsilon }$ weeks which was assumed to be the same for this cohort. $\sigma_{\delta ,1.5\;\text{T}}=16.4\;\text{ms}$ was estimated by averaging the T2* standard deviations of these age bins.

## 3 Results

**Fig. 2:**
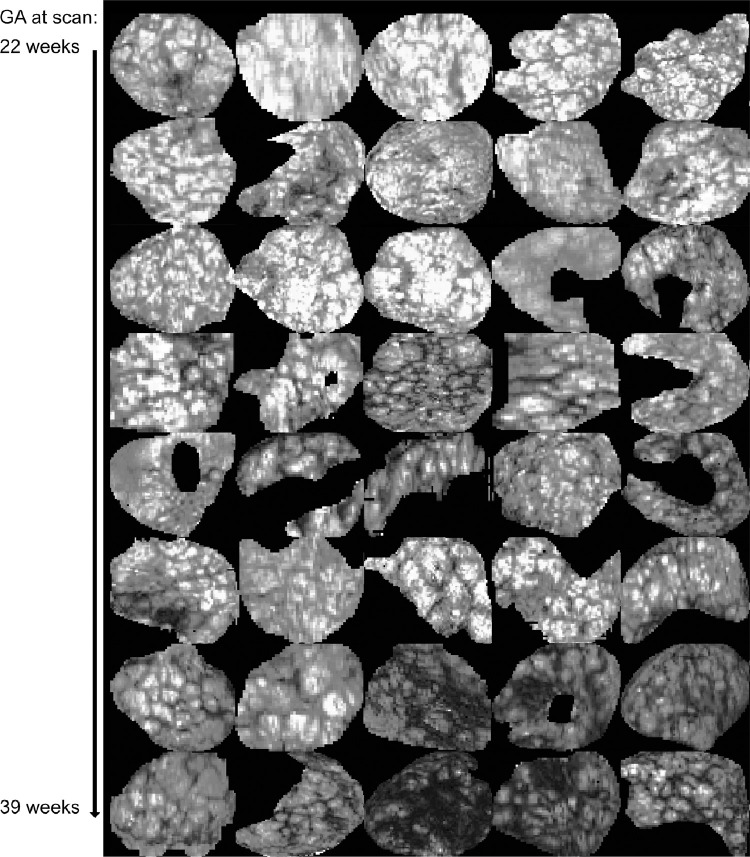
Illustration of central slices from segmented placental T2* maps for 40 low-risk cases used in this study. Images are sorted by GA (row-major).

We present results for all steps of the pipeline in the order of the processing steps. First, representative T2* data sets obtained with the sub-30 second whole placenta acquisition are illustrated in Fig. 2 ordered by GA. Second, the results of the segmentation step are presented and quantified and finally, the placental health prediction step is evaluated.

### 3.1 Segmentation

**Fig. 3:**
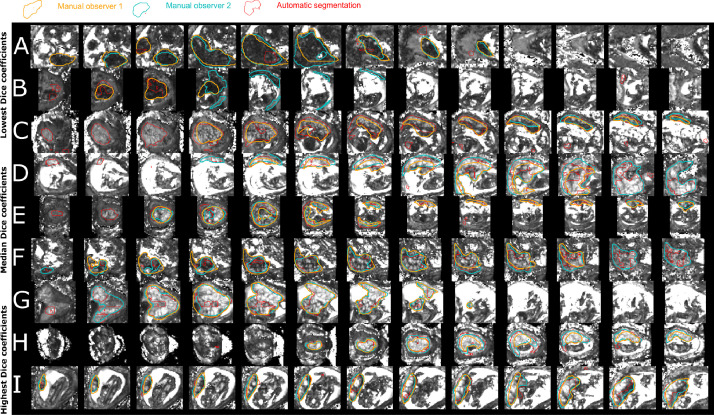
T2* maps and segmentations from both manual (blue and orange) and automatic (red) segmentations for nine cases for which all three segmentations were available. These include the three cases with the lowest Dice coefficients for the automatic segmentation (A-C), three cases close to the median Dice score (D-F) and the three best cases (G-I). For discussion see Section 3.1. (For interpretation of the references to colour in this figure legend, the reader is referred to the web version of this article.)

To evaluate the performance of the automatic segmentations, we compared the U-net segmentations obtained on the test sets of the cross-validation training scheme against one (randomly chosen) human expert segmentation. Automatic segmentations for nine cases chosen among the cases for which two manual segmentations were available, are shown in Fig. 3. Rows A–C depict three cases with the lowest Dice coefficients between automatic and manual segmentations, D–F with approximately the median Dice coefficients and rows G–I, cases with the three highest Dice coefficients. Cases A and B are of exceptionally low image quality and low SNR. In C, the first shown three slices are indeed placental tissue correctly classified by the automatic segmentation method but missed by both manual segmenters.

**Fig. 4:**
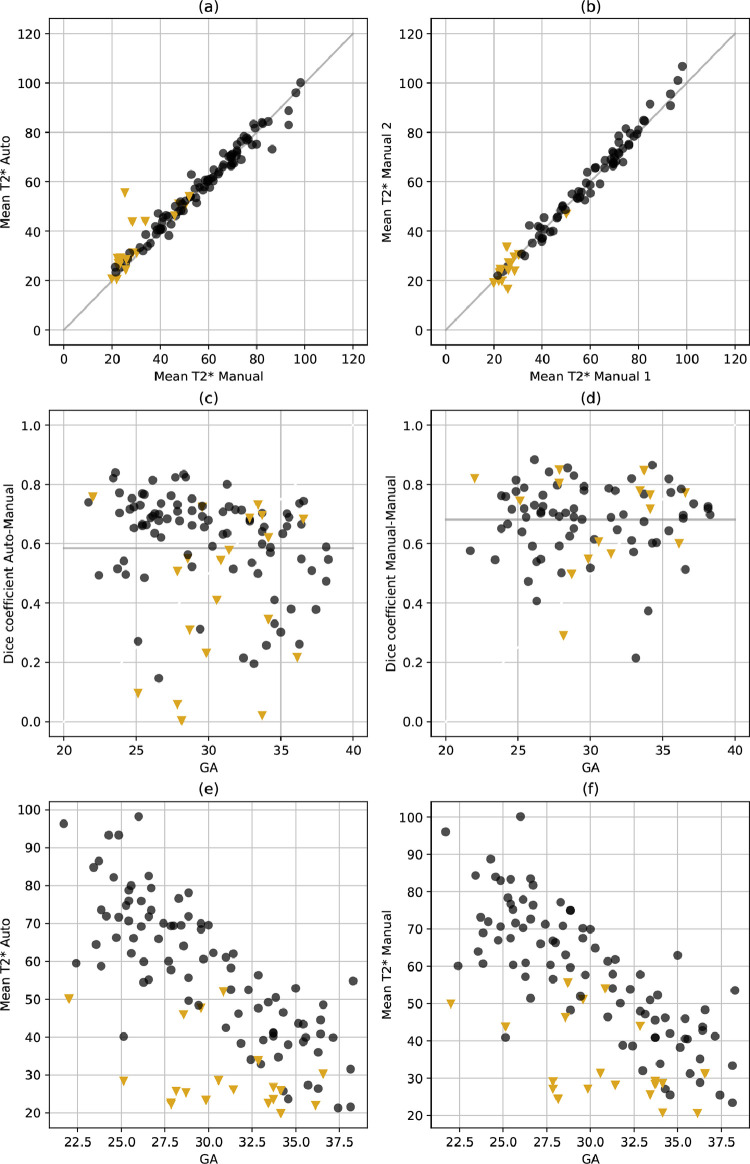
Quantitative results for the automatic segmentation. The results from the automatic segmentation in comparison with the one randomly chosen manual segmentation are given in the left column (a,c,e) and the results from the manual observers in the right column (b,d,f). (a) and (b) show the correspondence in mean T2* with the gray identity line. Plots (c) and (d) compare the Dice coefficient for all participants and (e) and (f) the mean T2* over GA. The gray lines in (c-d) illustrate the mean Dice coefficients of the automatic versus manual 0.58, n=108) and inter-rater (0.68, n=84) comparison. As before, the results from the normal cohort are shown in black and the results from the abnormal cohort with mustard triangles. Units: GA in weeks, T2* in ms.

Fig. 4 shows a quantitative comparison of segmentation results (Dice coefficients) and agreement in derived mean T2* values for the automatic segmentation compared to a randomly chosen manual segmentation and for the two expert annotators where data from both were available. The human inter-rater agreement measured as the Dice coefficient is higher but overall comparable to the agreement between automatic and randomly chosen manual segmentations (c-d). The mean Dice coefficient averaged over all available data for the automatic segmentation method is 0.58 and 0.68 for the inter-rater assessment (n=84). The disagreement between human and automated segmentations tends to be higher than that across the human observers. However, there is a good correspondence of the mean T2* results between automatic and manual segmentations (a) and between expert annotators (b) indicating that the segmentation performance is sufficient to derive meaningful T2* values. The Pearson correlation coefficients for inter-rater and automatic segmentation performance is 0.987 and 0.884 respectively, and the root mean squared error equals 3.2 and 7.4 ms respectively. To determine the effect of disagreements between masks, the mean T2* estimated in the full mask and that estimated in the area of agreement (intersection) or disagreement (xor) were plotted for the automatic versus manual and inter-rater comparison (Supplementary Figure 13). Additionally, the relation between Dice coefficient and mean T2* were plotted (Supplementary Figure 13).

We further visually assessed the manual segmentations in detail. Differences, where present, were usually detected at the placental-myometrium boundary or in scans with reduced image quality due to maternal habitus or posterior located placenta (see Fig. 3).

### 3.2 Placental health prediction

**Fig. 5:**
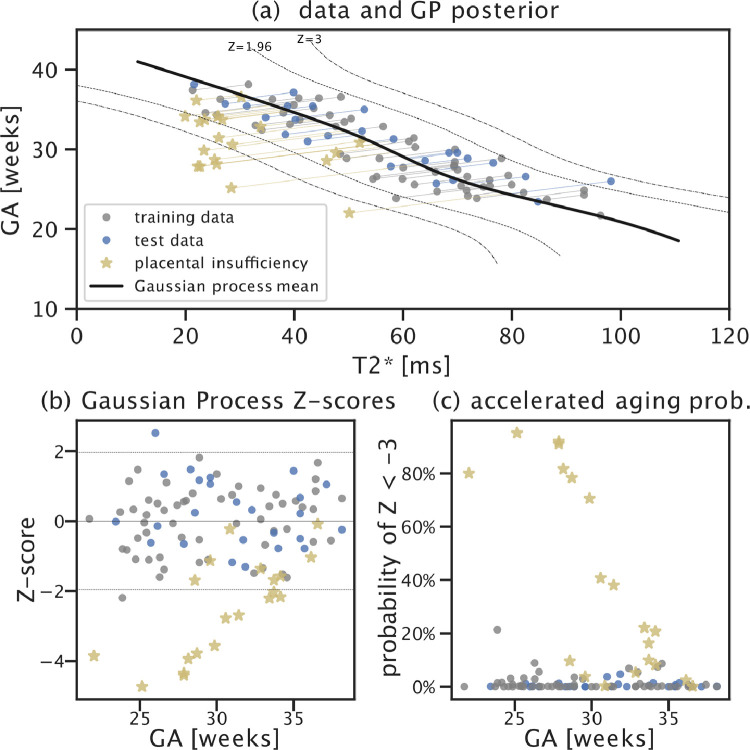
Total least squares Gaussian process placental health prediction results. (a) Posterior mean and 95% (Z=±1.96) and 99.7% (Z=±3) credible intervals (dashed lines) estimated from the training data (gray). Test low-risk data and the high-risk data are shown in blue and yellow. Residuals are indicated using lines projecting data onto the posterior mean. (b) Scatter plot of Z-scores vs gestational age show no age bias in training and test data, high-risk cases tend to have reduced Z-scores indicating lower biological mean T2* or lower biological GA than expected. (c) Probability of accelerated aging for each observed data point taking the Gaussian process credibility interval as well as uncertainty estimates of biological GA and mean T2* measurements into account. For least-squares fit and Gaussian process results based on manual segmentations see Supplementary Figures 9 and 10. (For interpretation of the references to colour in this figure legend, the reader is referred to the web version of this article.)

*Placental biological age and health prediction* The total least squares Gaussian process regression model (transformed back into GA vs mean T2* space) shows the expected approximately linear relationship between the two quantities (Fig. 5(a)). Both training and test data lie within the expected Z-score range ±3 (Fig. 5(b)), indicating that data from the normal cohort is indeed modelled as such. Furthermore, there is no discernible age-dependency in the Z-scores of the normal cohort. This is a result of the total least squares fit as demonstrated in Supplementary Fig. 9, where the Z-scores of an (OLS) model that was fit to predict biological placental age from mean T2* measurements shows a clear age-dependency in the training and test data (Supplementary Fig. 9(b)). This age bias of the (OLS) model causes erroneously elevated Z-scores for high-GA cases which reduces predictive power of accelerated aging in the high-risk cohort (see bottom of Fig. 10).

Utilizing a total least squares model, trained and evaluated on either manual or automatic segmentations, yields comparable predicted Z-scores for the low-risk cohort (±1.96SD[−0.64,0.56]) and no discernible age bias (see Bland Altman plots in Fig. 10). The abnormal cases exhibit similar bias and spread except for two to three cases, for which automatic segmentation-based Z-scores indicate higher abnormality than obtained via the manual segmentation. Hence, the predictions using automatic segmentations yield similar performance in the low-risk cohort and increase the rate of detected accelerated aging in the high-risk cohort.

**Fig. 6:**
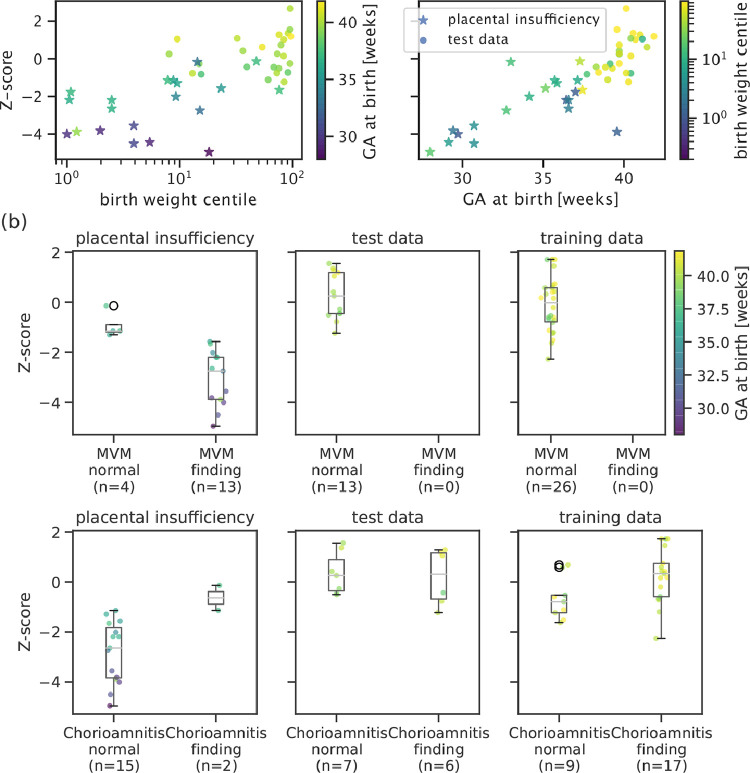
Top: The placental health Z-scores for the high-risk cohort and the low-risk test data are strongly related with degree of prematurity measured as GA at birth (right) and extremely low birth weight centile (left). Bottom: Z-scores grouped by histopathological examination results for training, test, and placental insufficiency data, colour-coded by GA at birth. Participants with maternal vascular malperfusion (MVM) exhibit lower Z-scores and belong to the group delivered prematurely. Chorioamnionitis does not affect Z-scores in control cases but seems to correlate to normal Z-scores in two of the high-risk participants.

*Z-score correlates with biological and histopathological information* The obtained Z-scores are further analyzed and depicted in Fig. 6. A positive linear trend between Z-scores and both GA at birth and the logarithm of the birth weight centile can be observed in (a). Large deviations from the trends (Z-score <4) are associated with either premature birth (GA at birth 28 weeks) or very low birth weight centile (<1st centile). Correlation between Z-score and available histopathology findings are shown in Fig. 6(b). In high-risk cases, lower Z-scores are associated with maternal vascular malperfusion (MVM) while signs of chorioamnionitis coincides with normal Z-scores. No cases with MVM have been found in the analysed low-risk cohort, and the Z-scores of the cases with chorioamnionitis findings in the low-risk cohort obtain similar (normal) Z-scores. No clear relationship between maternal age or BMI and Z-scores were found in the control cohort (see Figure 12). However, a correlation between advanced maternal age and Z-score can be observed in the placental insufficiency group.

**Fig. 7:**
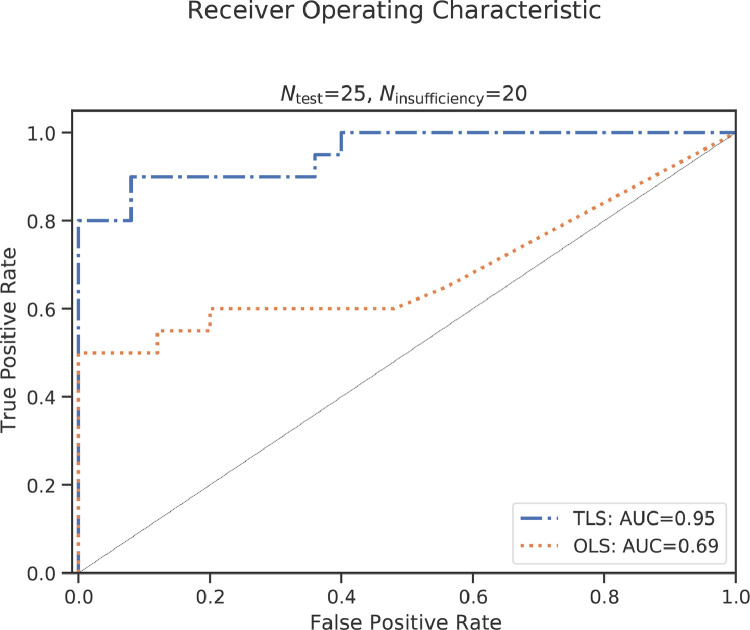
The receiver operating characteristic curve using the accelerated aging probability to predict ”placental insufficiency” in the combined test and placental insufficiency groups. The total least squares (TLS) and ordinary least squares (OLS) version have an area under the curve (AUC) of 0.95 and 0.69, respectively..

The result of the receiver operator characteristic evaluation quantifying the predictive potential of the proposed maturation assessment is shown in Fig. 7. The proposed TLS-based pipeline has an AUC of 0.95. Using an OLS-based approach yields an AUC of 0.69.

### 3.3 Generalization and 1.5 T data

The results from the 1.5 T data in Supplementary Figure 11 illustrate the same relationships between placental parameters and clinical findings as for the 3 T data. The subjects with PE have consistently reduced Z-scores (Z<−1.5, Fig. 11(b)) and elevated probability of abnormally accelerated aging (Fig. 11(c)).

## 4 Discussion and conclusion

This study presents a fully automatized pipeline to assess the maturation of a placenta in-vivo from a sub-30 second MRI scan. The proposed two-step process, consisting of first, a fully automatic segmentation and second, the assessment of placental age and health, assures accessibility and interpretability. The availability of the whole organ segmentations as an intermediate step allows visual inspection and effortless extension to other quantitative markers. Its potential for direct translation is illustrated by including results from a different cohort, scanned on a different scanner with lower field strength and altered acquisition parameters (Fig. 11).

The mean T2* values obtained from the automatic segmentations (Fig. 4) decay over GA in line with literature values (Sørensen, Peters, Fründ, Lingman, Christiansen, Uldbjerg, 2013, Sørensen, Hutter, Seed, Grant, Gowland, 2020, Sinding, Peters, Frøkjær, Christiansen, Petersen, Uldbjerg, Sørensen, 2017, Abaci Turk, Abulnaga, Luo, Stout, Feldman, Turk, Gagoski, Wald, Adalsteinsson, Roberts, Bibbo, Robinson, Golland, Grant, Barth, 2020), and are reduced as previously observed in high-risk cases (Sinding, Peters, Poulsen, Frøkjær, Christiansen, Petersen, Uldbjerg, Sørensen, 2018, Ho, Hutter, Jackson, Seed, McCabe, Al-Adnani, Marnerides, George, Story, Hajnal, Rutherford, Chappell, 2020). The obtained placental Z-scores for the high-risk cohort with apparent placental insufficiency correlate well with outcome assessed by birth weight centile, GA at birth and histopathological results (Fig. 6). In addition, reduced Z-scores, hypothesized to relate to accelerated aging, corresponded to the presence of maternal vascular malperfusion in this study. This is in line with the most common components of MVM, villous infarction, retroplacental hemorrhage, accelerated villous maturation, and distal villous hypoplasia, often occuring in placentas with long periods of fetal hypoxia, often co-occuring with other signs such as of accelerated villous maturation (Ernst, 2018, Parks and Catov, 2020). Signs of chorioamnionitis were found equally in our low and high-risk cohorts. However, in our high-risk cohort the presence of chorioamnionitis was associated with normal Z-scores, suggesting different pathological processes in these high-risk cases such as a more acute pathology associated with inflammation.

T2* constitutes an indirect measure of placental oxygenation via the BOLD effect as described above and the T2* values are influenced by a range of both acquisition-related factors such as maternal habitus, placental location and coil placement as well as intrinsic factors such as hematocrit values, characteristics of hemoglobin, maternal and fetal blood fractions and geometric effects among many others. These factors influence the T2* not through the BOLD effect but are nevertheless captured by the Gaussian process regression model either contributing to the model uncertainty or to the mean T2* age trend, depending on the effects’ relation with placental maturation and aging.

Automatic segmentation of the placenta has attracted reasonable interest mainly on anatomical data with the aim to perform volumetrics (Alansary, Kamnitsas, Davidson, Khlebnikov, Rajchl, Malamateniou, Rutherford, Hajnal, Glocker, Rueckert, Kainz, 2016, Han, Bao, Sun, Wen, Xia, Zhao, Du, Yan, 2019, Wang, Zuluaga, Pratt, Aertsen, David, Deprest, Vercauteren, Ourselin, 2015). Previous functional T2*-based studies have used manual segmentations of either individual slices (Sørensen, Peters, Fründ, Lingman, Christiansen, Uldbjerg, 2013, Sørensen, Hutter, Seed, Grant, Gowland, 2020, Sinding, Peters, Frøkjaer, Christiansen, Petersen, Uldbjerg, Sørensen, 2016) or the entire organ (Abaci Turk, Abulnaga, Luo, Stout, Feldman, Turk, Gagoski, Wald, Adalsteinsson, Roberts, Bibbo, Robinson, Golland, Grant, Barth, 2020, Hutter, Slator, Jackson, Gomes, Ho, Story, O’Muircheartaigh, Teixeira, Chappell, Alexander, Rutherford, Hajnal, 2018). The presented study allows automatic segmentation of the entire placenta, but does not provide exact volumetrics due the distortions, inter-slice motion present in the Multi-Echo Gradient Echo data, as well as the relatively low Dice coefficients even between manual segmentations especially for the cases of placental insufficiency.

The aim of this pipeline was to establish a fully automatic assessment, close to the data and accessible throughout the processing pipeline. However, this could be expanded with further post-processing steps. These include placental 3D reconstruction, i.e. using orthogonal slice stacks or multiple dynamics as has been recently proposed for T2* data of the brain (Blazejewska et al., 2017) and the placenta (Uus et al., 2020) or inter-slice motion correction techniques (Luo et al., 2017) to improve the 3D continuity of the data thus enabling 3D patch-based segmentation and/or placental flattening techniques (Abulnaga, Abaci Turk, Bessmeltsev, Grant, Solomon, Golland, 2019, Miao, Mistelbauer, Karimov, Alansary, Davidson, Lloyd, Damodaram, Story, Hutter, Hajnal, Rutherford, Preim, Kainz, Groller, 2017), resulting in representations in a common coordinate system.

This study deliberately proposes a pipeline built explicitly to be close to the original and wide-spread 2D multi-slice acquisition which makes it extendable to other applications of quantification of T2* data. It provides the full organ segmentation for all acquired slices and this can be expanded at any stage by reconstruction and registration approaches. Similarly, the proposed maturation quantification method is independent of the actual measurement method of the T2* values. For the evaluation performed here, mean T2* over the whole organ was chosen as this is the most widely used measure. It thus provides excellent validation that the quality of the segmentation reaches the requirements to identify pathological cases and the new maturation assessment concept: The obtained mean T2* values as a function of age matches observations in the recent literature (Sørensen, Peters, Fründ, Lingman, Christiansen, Uldbjerg, 2013, Sørensen, Hutter, Seed, Grant, Gowland, 2020). Further measures of interest for placental characterisation, including spatial information as previously proposed histogram based measures and texture analysis (Lo, Roberts, Schabel, Wang, Morgan, Liu, Studholme, Kroenke, Frias, 2018, Hirsch, Roberts, Grigsby, Haese, Schabel, Wang, Lo, Liu, Kroenke, Smith, Kelleher, Broeckel, Kreklywich, Parkins, Denton, Smith, DeFilippis, Messer, Nelson, Hennebold, Grafe, Colgin, Lewis, Ducore, Swanson, Legasse, Axthelm, MacAllister, Moses, Morgan, Frias, Streblow, 2018, Hutter, Slator, Jackson, Gomes, Ho, Story, O’Muircheartaigh, Teixeira, Chappell, Alexander, Rutherford, Hajnal, 2018), can be included in future work. Another possible directly supported step would be the use of a convolutional neural network on the imaging data itself to map image properties against age instead of the mean T2* values.

The available data including comprehensive clinical information about maternal and fetal outcome has allowed differentiation between low- and high-risk cohorts, with evidence to support placental insufficiency in the latter. The low-risk cohort is crucial to train the prediction algorithm as it allows the reasonable assumption that the chronological age is an unbiased estimator of biological age. Histopathological evaluation was performed in fewer than 50% of the cases, which means that we can not exclude that individual cases of placental pathological findings exist in the low-risk cohort. Given the other available outcome parameters, including the need for each low risk case to have resulted in a term-born neonate of appropriate weight without maternal and fetal complications, we are however confident that these cases represent indeed “normal” placental maturation.

The segmentation U-net training requires, for clinical settings, a relatively large training set. The data used in this study were collected from a large scale study and presents over 100 datasets from in-vivo placental MRI scans together with comprehensive outcome information allowing the creation of a well characterized normal cohort, which is one of the largest such data collections. Rigorous selection of control cases was performed, only considering cases with complete outcome and no history of fetal or maternal complications (see section “Data”). However, for the high-risk cohort with suspected “placental insufficiency”, cases with either PE or fetal growth restriction have been analysed jointly to reach sufficient numbers. It is appreciated though that placental pathology may be different in these two groups and also between cases with early versus late onset PE. The PE cases were explored in a previous publication (Ho et al., 2020). The high-risk cohort in the 1.5 T data was more uniform in the pathology as all were clinically diagnosed with PE, which was well reflected in the clearer separation in Z-scores.

Compared with studies of other organs, the inter-rater agreements measured as Dice coefficients were relatively low. Retrospective analysis of the segmentations reveals that especially scans with low SNR such as common in posterior placentas, subjects with high BMI, and the presence of pathology (Fig. 3A and B) lead to increased inter-rater disagreement. In multiple cases, the automatic segmentations included areas missed in one or in both expert segmentations. Exemplary cases are shown in Fig. 3C,E and F. Important for the proposed pipeline is the ability to robustly extract meaningful mean T2* values to characterise placental health. Comparison of the mean T2* evaluated in the full mask and in the areas of agreement and disagreement between raters, as displayed in Supporting Figure 13, showed that the mean T2* is relatively invariant to the contributions from areas of disagreement even for cases where the overlap was small as depicted for example in Fig. 3A and B. Although not critical for mean T2* measurements, future studies can explore the benefit of a neural network architecture and training method search or the benefit of additional MRI contrasts.

Neither BMI nor maternal age appear related to the predicted Z-scores in the control cohort. We speculate that the proposed pipeline is not affected by the decreased image quality in participants with higher BMI. However, there was a correlation between maternal age and prediction in the high risk cohort as well as an increased mean BMI and maternal age in the high- compared with the low-risk cohort. This matches with the known increased risk for eg. PE both with increased BMI (Lean et al., 2017) and maternal BMI (Sohlberg et al., 2012).

The available datasets are not uniformly distributed over GA with a specifically reduced occurrence before 22 weeks and most notably after 36 weeks. This both influences the ability of the segmentation U-net to identify placental tissue in later pregnancy (see decay in Dice coefficients over GA) and the relatively wide credibility interval of the Gaussian process model for representations with lower mean T2* as would normally occur in later GA and in high-risk cases with later confirmed placental insufficiency. For our cohorts, the predictive power of the pipeline is likely limited by a reduced density of normative data in the age extremes. In particular, lacking a meaningful sample of older placental insufficiency cases, we can not reliably assess the predictive performance of detecting accelerated aging in late gestation (compare Fig. 5). Assuming a constant severity of placental abnormality (irrespective of age) in our data, the distance between placental insufficiency and normative data appears to shrink with age, potentially disappearing after 34 weeks. Future data collection and analysis should determine the clinical utility of the proposed method for cases above 34 weeks.

In addition, recruiting and scanning cases with suspected placental insufficiency is constrained by the challenges of clinical instability, need for urgent early delivery and the availability of scan slots. The reduced Dice coefficients for this cohort might again result from a lack of training samples for such placentas. The strength of the proposed pipeline is that it keeps the clinician in the loop and allows manual intervention via access to the T2* maps and segmentation masks.

Finally, if the achieved maturation marker is to be used in longitudinal studies to evaluate e.g the influence of treatments, its robustness to track individual pregnancies over time needs to be established with longitudinal data.

This study proposes a comprehensive, automatic pipeline to evaluate the maturation of a placenta in vivo from a short T2* MRI scan. Further factors which could be studied using this suggested pipeline include the effects and alteration of maturation in prolonged pregnancies or gestational diabetes. Inclusion into the clinical workflow is facilitated by the ease of use and the transparent steps employed.

## CRediT authorship contribution statement

**Maximilian Pietsch:** Methodology, Writing - original draft, Software, Supervision. **Alison Ho:** Data curation, Investigation. **Alessia Bardanzellu:** Investigation. **Aya Mutaz Ahmad Zeidan:** Investigation. **Lucy C. Chappell:** Investigation, Supervision, Resources. **Joseph V. Hajnal:** Supervision, Resources, Funding acquisition. **Mary Rutherford:** Supervision, Resources, Funding acquisition, Conceptualization. **Jana Hutter:** Conceptualization, Methodology, Writing - original draft, Software, Funding acquisition, Supervision.

## Declaration of Competing Interest

Authors declare that they have no conflict of interest.

## Appendix A Supplementary materials

Supplementary Data S1 Supplementary Raw Research Data. This is open data under the CC BY license http://creativecommons.org/licenses/by/4.0/ Supplementary Data S1

## Footnotes

1 https://github.com/MIC-DKFZ/batchgenerators
