# Supplementary material for: APPLAUSE: Automatic Prediction of PLAcental health via U-net Segmentation and statistical Evaluation
Source: Med Image Anal. 2021 Aug;72:102145. doi: 10.1016/j.media.2021.102145 (PMC8350147; doi:10.1016/j.media.2021.102145)
Supplement: Supplementary Data S1 — Supplementary Raw Research Data. This is open data under the CC BY license http://creativecommons.org/licenses/by/4.0/ [file mmc1.pdf]

Supplementary Material

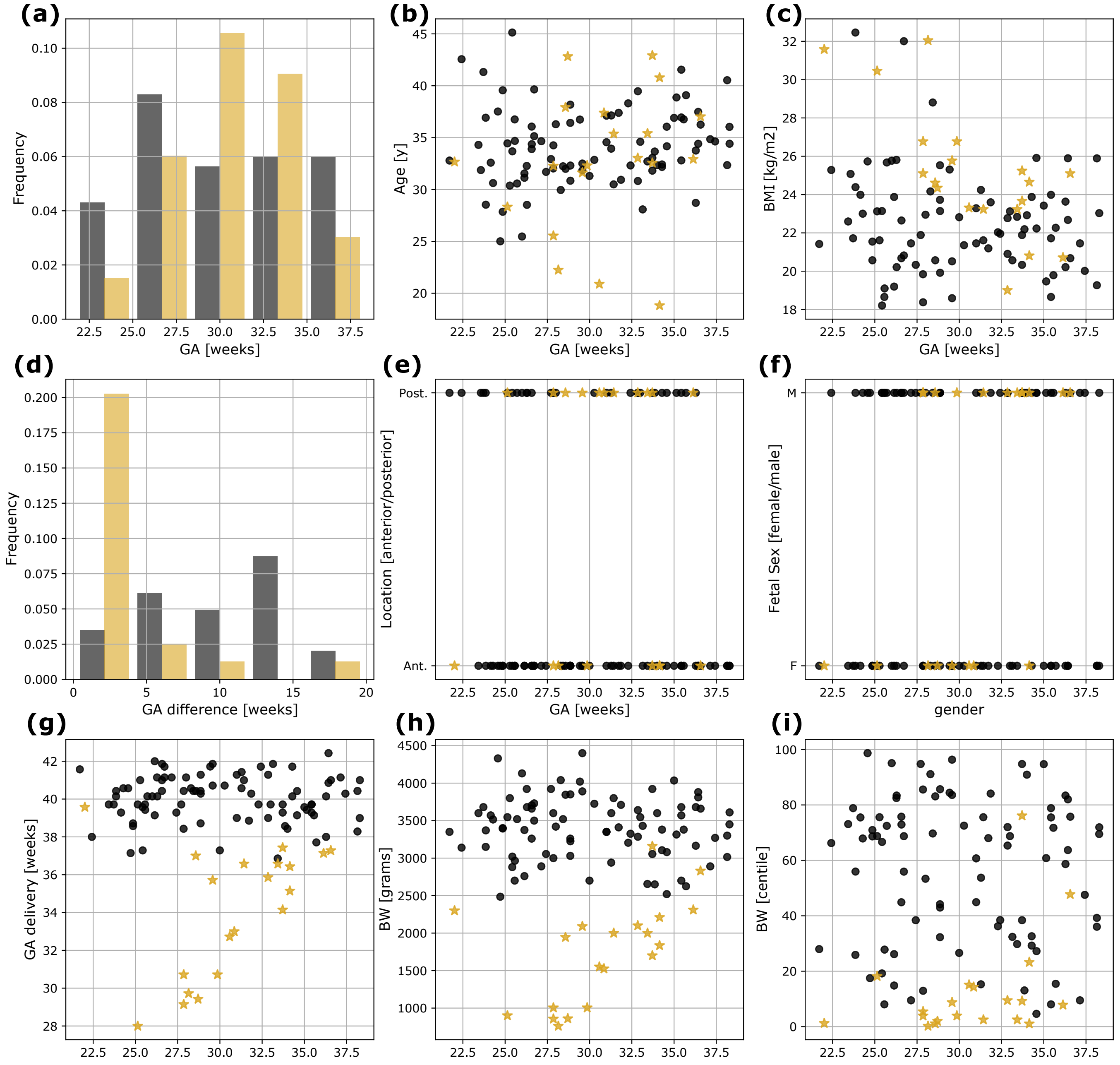

Supplementary Figure 8. The demographics of the cohort are graphically illustrated with the control cohort in gray and the abnormal cohort in mustard. (a) Distribution of the GA at the time of scan, maternal age (b), and (c) BMI at the time of scan, distribution of the difference from GA at scan to GA at birth (d), placental location (e) and fetal sex (f) distribution. Finally, gestation at birth (g), birth weight (BW) in gram (h) and birth weight centile (i).

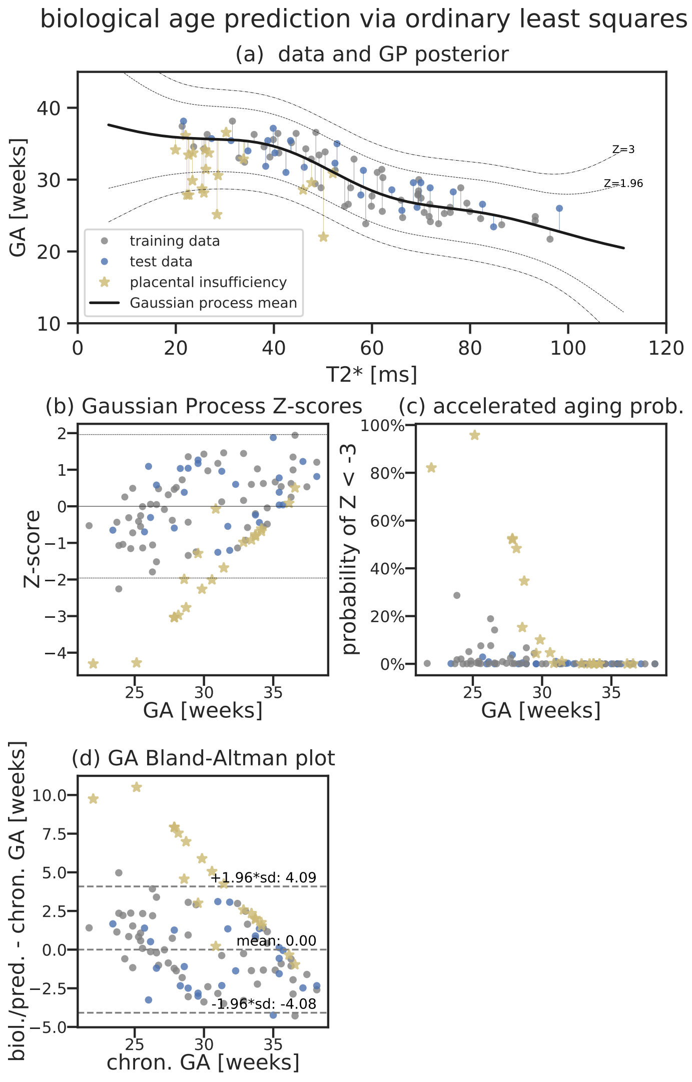

Supplementary Figure 9. Ordinary least squares (OLS) Gaussian Process results. This fit was performed with identical initial hyperparameters, fitting procedure and data as in Fig. 5 but using an ordinary least squares fit instead of the total least squares projection (TLS). Here, Z-scores are derived from the difference between chronological and predicted biological GA (b). The clear age-trend in the residuals for training and testing data indicate that an error-in-variable model is required.

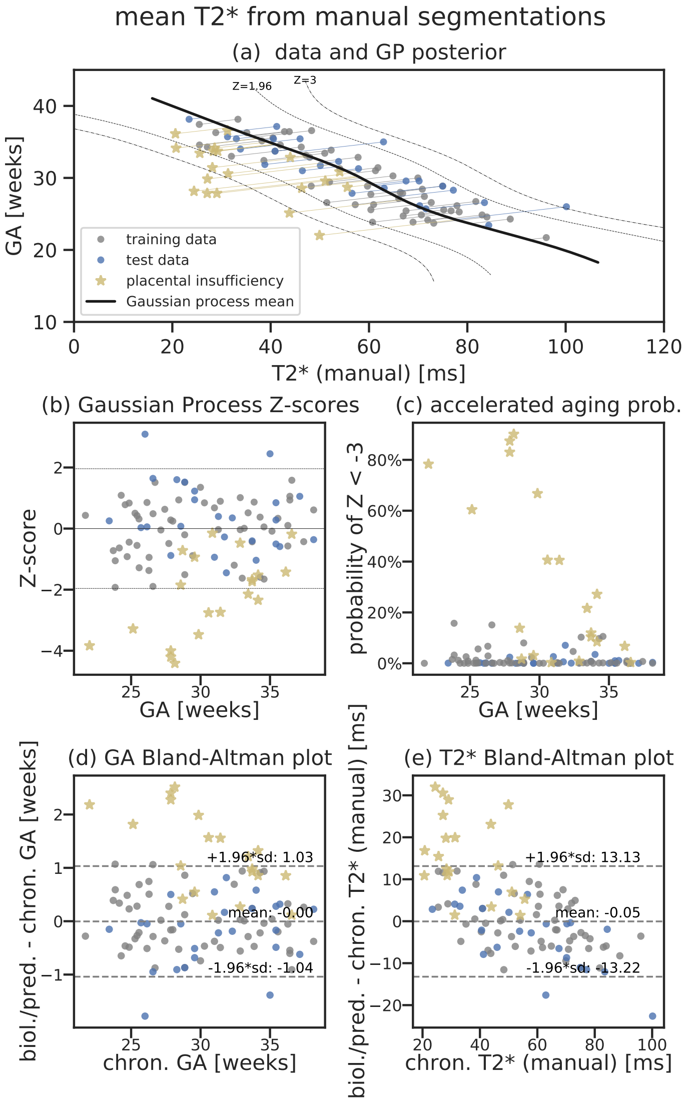

Supplementary Figure 10. Equivalent of Fig. 5 using manual segmentations for T2* estimation.

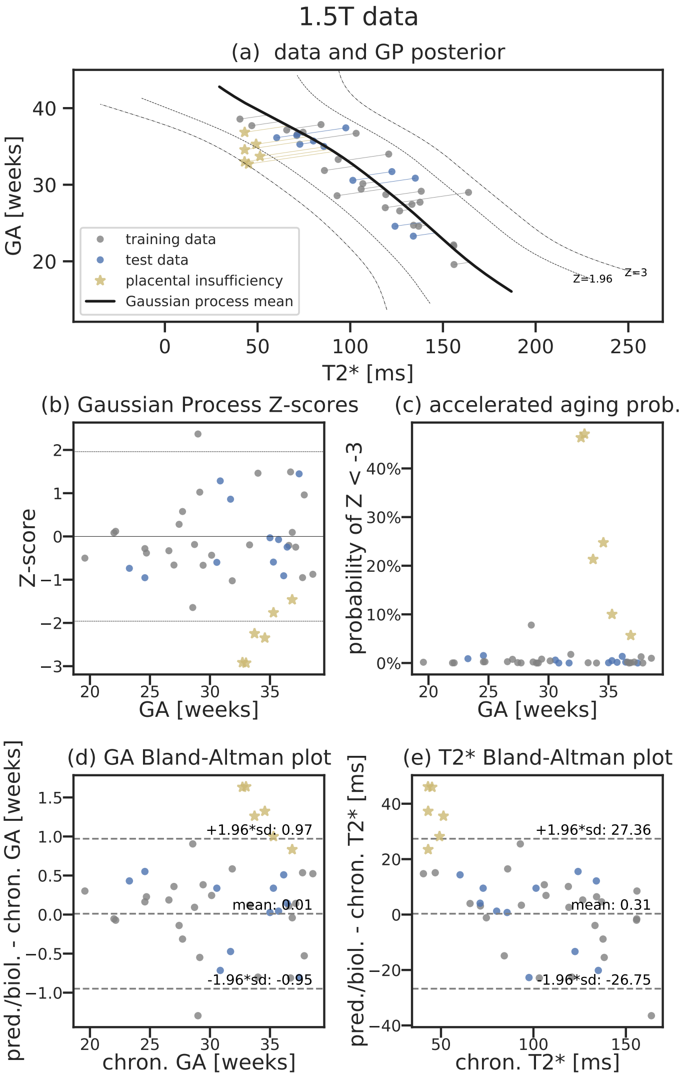

Supplementary Figure 11. Equivalent of Fig. 5 using 1.5T data.

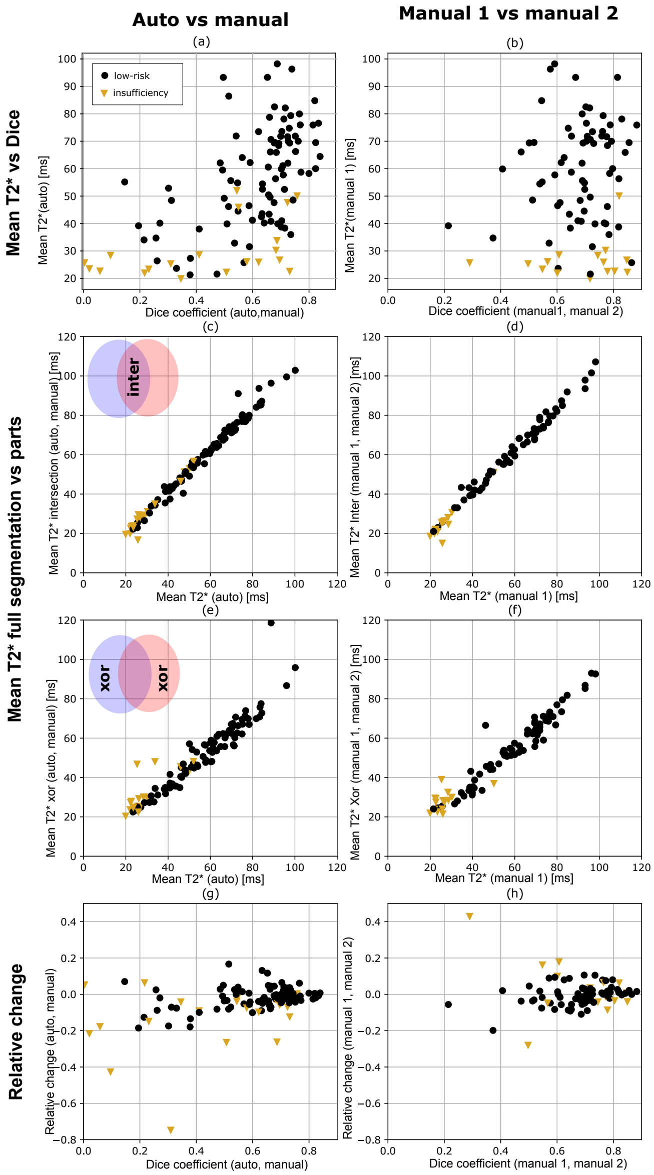

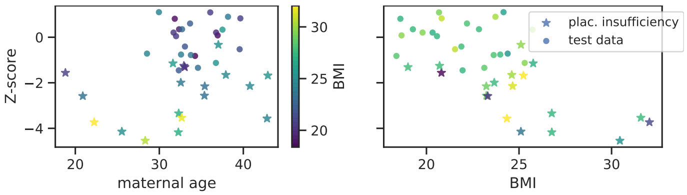

Supplementary Figure 12. The placental health Z-scores for the high-risk cohort and the low-risk test data versus maternal age (left) and maternal BMI (right).

Supplementary Figure 13. The effect of the segmentation on the obtained T2* measures. The left column (a,c,e,g) compares the measures between automatic and fixed randomly chosen manual segmentations. Plots on the right (b,d,f,h) compare both manual segmentations. (a-b) show the mean T2* plotted against the Dice coefficient, (c-f) the mean T2* obtained from the entire segmentation (x-axis) plotted against the mean T2* in the (c-d) intersection of both segmentations or (e-f) in the area where no overlap was achieved. Finally plots of the relative T2* change using manual and manual 1 as references are shown against the Dice coefficients in (g-h).
